# Comparison of the Sulfonamide Inhibition Profiles of the β- and γ-Carbonic Anhydrases from the Pathogenic Bacterium *Burkholderia pseudomallei*

**DOI:** 10.3390/molecules22030421

**Published:** 2017-03-07

**Authors:** Daniela Vullo, Sonia Del Prete, Pietro Di Fonzo, Vincenzo Carginale, W. Alexander Donald, Claudiu T. Supuran, Clemente Capasso

**Affiliations:** 1 Laboratorio di Chimica Bioinorganica, Dipartimento Di Chimica, Università degli Studi di Firenze, Polo Scientifico, Via della Lastruccia 3, 50019 Sesto Fiorentino, Florence, Italy; 2 Istituto di Bioscienze e Biorisorse, CNR, Via Pietro Castellino 111, 80131 Napoli, Italy; sonia.delprete@unifi.it (S.D.P.); pidifo@gmail.com (P.D.F.); vincenzo.carginale@cnr.it (V.C.); 3 Sezione di Scienze Farmaceutiche e Nutraceutiche, Dipartimento Neurofarba, Università degli Studi di Firenze, Via U. Schiff 6, 50019 Sesto Fiorentino, Florence, Italy; claudiu.supuran@unifi.it; 4 School of Chemistry, University of New South Wales, Sydney, New South Wales 2052, Australia; w.donald@unsw.edu.au

**Keywords:** carbonic anhydrase, metalloenzymes, pathogens, β-class, sulfonamide, *Burkholderia pseudomallei*

## Abstract

We have cloned, purified, and characterized a β-carbonic anhydrase (CA, EC 4.2.1.1), BpsCAβ, from the pathogenic bacterium *Burkholderia pseudomallei*, responsible for the tropical disease melioidosis. The enzyme showed high catalytic activity for the physiologic CO_2_ hydration reaction to bicarbonate and protons, with the following kinetic parameters: k_cat_ of 1.6 × 10^5^ s^−1^ and k_cat_/K_M_ of 3.4 × 10^7^ M^−1^ s^−1^. An inhibition study with a panel of 38 sulfonamides and one sulfamate—including 15 compounds that are used clinically—revealed an interesting structure–activity relationship for the interaction of this enzyme with these inhibitors. Many simple sulfonamides and clinically used agents such as topiramate, sulpiride, celecoxib, valdecoxib, and sulthiame were ineffective BpsCAβ inhibitors (K_I_ > 50 µM). Other drugs, such as ethoxzolamide, dorzolamide, brinzolamide, zonisamide, indisulam, and hydrochlorothiazide were moderately potent micromolar inhibitors. The best inhibition was observed with benzene-1,3-disulfonamides—benzolamide and its analogs acetazolamide and methazolamide—which showed K_I_ in the range of 185–745 nM. The inhibition profile of BpsCAβ is very different from that of the γ-class enzyme from the same pathogen, BpsCAγ. Thus, identifying compounds that would effectively interact with both enzymes is relatively challenging. However, benzolamide was one of the best inhibitors of both of these CAs with K_I_ of 653 and 185 nM, respectively, making it an interesting lead compound for the design of more effective agents, which may be useful tools for understanding the pathogenicity of this bacterium.

## 1. Introduction

**Figure 1:**
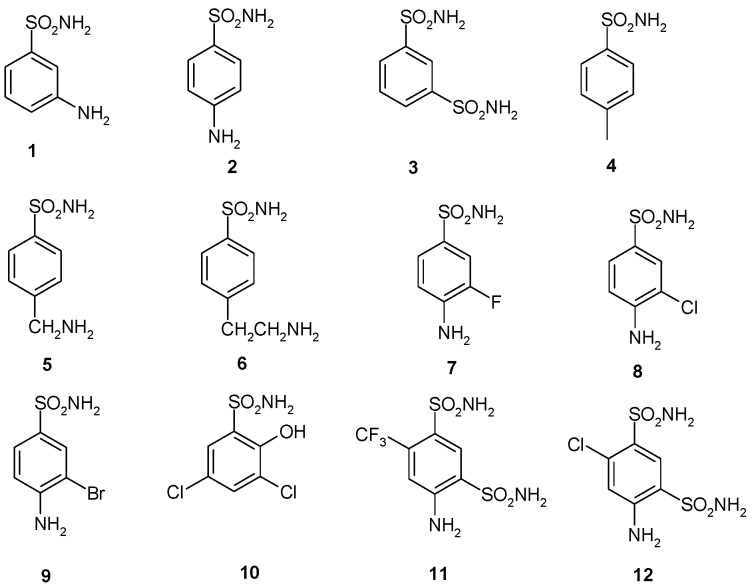

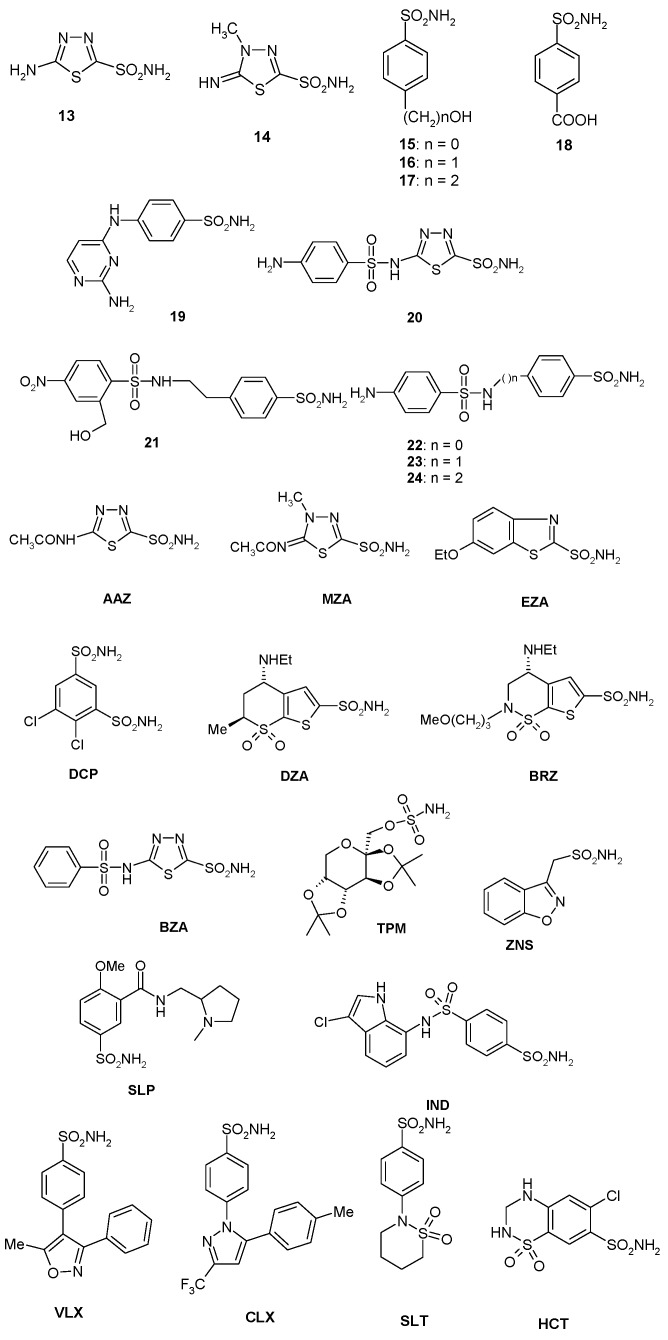
Structure of sulfonamides/sulfamates investigated in the present study. AAZ: acetazolamide; BRZ: brinzolamide; BZA: benzolamide; CLX: celecoxib; DCP: dichlorophenamide; DZA: dorzolamide; EZA: ethoxzolamide; HCT: hydrochlorothiazide; IND: indisulam; MZA: methazolamide; SLP: sulpiride; SLT: sulthiame; TPM: topiramate; VLX: valdecoxib; ZNS: zonisamide.

Our groups started to investigate the biochemical properties, kinetic constants and inhibition profiles of numerous classes of carbonic anhydrases (CAs; EC 4.2.1.1) in different species of pathogenic and non-pathogenic bacteria (see Table 1). CAs are ubiquitous metalloenzymes that catalyze the reversible hydration of carbon dioxide with the production of bicarbonate and protons. The CA superfamily includes seven distinct classes known as the α, β, γ, δ, ζ, η, and θ [1,2,3,4,5,6,7,8,9,10,11,12,13]. The α-, β-, δ-, η-, and perhaps θ-CAs use Zn(II) ions at the active site, the γ-CAs are probably Fe(II) enzymes (but they are also active with bound Zn(II) or Co(II) ions) [14,15,16,17,18,19,20,21], and the ζ-class CAs are cambialistic enzymes, active both with Cd(II) or Zn(II) bound within the active site in order to perform the physiologic reaction catalysis [22,23,24]. The metal ion from the CA active site is coordinated by three His residues in the α-, γ-, δ-, and probably θ-classes, by one His and two Cys residues in β- and ζ-CAs, or by two His and one Gln residues in the η-class, with the fourth ligand being a water molecule/hydroxide ion acting as nucleophile in the catalytic cycle of the enzyme [11,25,26,27,28,29,30]. All CAs identified in animal systems belong to α-class. CAs identified in plants and algae belong to the α-, β-, γ-, δ-, and θ-classes; fungi encode for α- and β-CAs; protozoa encode for α-, β-, or η-CAs. As shown in Table 1, bacteria encode for enzymes belonging to the α-, β-, and γ-CA classes [11,12,27,29,31,32,33]. Moreover, as described in literature, the genome of Gram-negative bacteria belonging to the genera *Buchnera* and *Rickettsia* does not encode for any CAs [29]. Thus, the distribution pattern of CAs in bacteria is very fascinating [29]. In the last year, it has been demonstrated in vivo that the inhibition of bacterial CAs influences the pathogenicity and/or the growth of the microorganism [27,34,35,36,37,38,39]. These promising data on live bacteria allow us to propose bacterial CAs inhibition as an approach for obtaining anti-infective agents with a new mechanism of action compared to classical antibiotics. At present, infectious diseases are the second-leading cause of death in the world, and the development of bacterial antibiotic-resistance is an inevitable and widespread phenomenon inherent to most drugs. Several classes of CA inhibitors (CAIs) are known to date, among which the metal complexing anions and sulfonamides and their bioisosteres are the most investigated for the inhibition of the bacterial CAs [12,40,41,42,43,44]. Anions such as the inorganic metal-complexing or more complicated species such as the carboxylates bind to CAs, but generally with less efficiency compared to the sulfonamides [45]. Anions may bind either the tetrahedral geometry of the metal ion or as trigonal–bipyramidal adducts [45]. The antibiotic revolution in medicine was represented by sulfonamides, which were the first antimicrobial drugs (Figure 1) [31,41]. Sulfonamide derivatives that were used clinically, including acetazolamide, methazolamide, ethoxzolamide, dichlorophenamide, dorzolamide, and brinzolamide, bind in a tetrahedral geometry to the Zn(II) ion in deprotonated state, with the nitrogen atom of the sulfonamide moiety coordinated to Zn(II) and an extended network of hydrogen bonds involving amino acid residues Thr199 and Glu106 (numbering system used for the human CA, isoform I) also participating in the anchoring of the inhibitor molecule to the metal ion [31,41,46]. The aromatic/heterocyclic part of the inhibitor interacts with hydrophilic and hydrophobic residues of the cavity. The protein fold in the diverse CA classes (seven CA-classes) is highly different [14,15,16,17,18,19,20,21], mainly due to their different oligomeric state. Successful inhibition studies with sulfonamides should allow the discovery of highly isoform-selective CAIs, which may lead to a new generation of drugs targeting these widespread enzymes.

In this context, we cloned, expressed, and purified the recombinant β-CA (BpsCAβ, Accession number: WP_004550949.1) identified in the genome of *Burkholderia pseudomallei*, a Gram-negative saprophytic bacteria responsible of melioidosis, which is an endemic disease of tropical and sub-tropical regions [47]. The *B. pseudomallei* genome encodes for only β- and γ-CAs. In the present manuscript, the inhibition profile of BpsCAβ was investigated using a wide series of sulfonamides/sulfamates. The inhibition profile of BpsCAβ was compared with those obtained for the human α-CAs (hCA I and hCAII) and the other bacterial BpsCAγ previously identified in the genome of *Burkholderia pseudomallei* [7] and investigated by our groups for its inhibition profiles with anions and sulfonamides. This study may be of interest for designing new types of inhibitors that may have clinical applications, because *Burkholderia pseudomallei* is fundamentally resistant to penicillin, ampicillin, first-generation and second-generation cephalosporins, macrolides, quinolones, and most aminoglycosides [48].

## 2. Results and Discussion

### 2.1. Purification and Protonographic Analysis

**Figure 2:**
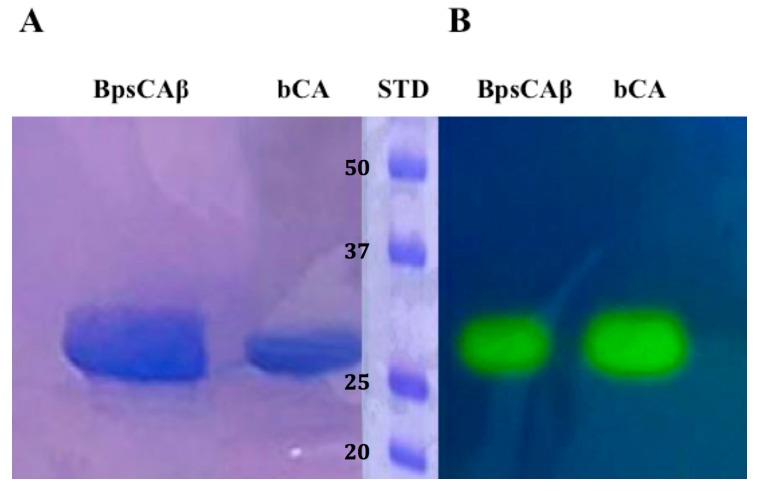
Comparison between SDS-PAGE and protonography of recombinant *B. pseudomallei* β-CA (BpsCAβ). (**A**) SDS-PAGE of bCA and BpsCAβ. The gel was stained with Coomassie blue; (**B**) Protonogram of α-CA from bovine erythrocytes (bCA) and BpsCAβ. The yellow bands on the blue background correspond to the bCA and BpsCAβ activity, which determine the drop of pH from 8.2 to the transition point of the dye (pH 6.8). Yellow bands were obtained with an incubation time of 15 s. STD: molecular markers starting from the top had the following molecular weights: 50 kDa, 37 kDa, 25 kDA, and 20 kDa (see bold numbers on the figure).

The recombinant BpsCAβ was heterologously expressed as soluble protein in the cytoplasm of the *E. coli* (DE3) codon plus cells and produced as a fusion protein containing a His-tag tail at its N-terminal amino acid sequence. After sonication and centrifugation, BpsCAβ was purified from the cell extract using the His-select HF Nickel column as the affinity column. The fusion protein showed an apparent molecular weight of about 30 kDa, as indicated by sodium dodecyl sulfate (SDS)-polyacrylamide gel electrophoresis (PAGE) (SDS-PAGE, Figure 2A). The apparent molecular weight of 30 kDa on the SDS-PAGE is due to the SDS concentration, which determines the separation of the subunits of the BpsCAβ. Generally, β-CAs catalyze the hydration of carbon dioxide to bicarbonate and protons when the β-CAs monomers assemble into dimers, tetramers, or octamers. To investigate the hydratase activity of BpsCAβ and bCA (commercial α-CA from bovine erythrocytes, purchased from Sigma) on the SDS-PAGE gel, samples of BpsCAβ or bCA were prepared and loaded on the gel. The protonography technique is based on the monitoring of pH variation in the gel due to the catalyzed conversion of CO_2_ to bicarbonate and protons. The production of hydrogen ions during the CO_2_ hydration reaction due to the bCA or BpsCAβ hydratase activity lowers the pH of the solution until the color transition point of the dye (bromothymol blue) is reached (pH 6.8) [79,80,81]. This dye appears blue in its deprotonated form, while its color changes to yellow in the protonated form. The protonated form of bromothymol blue has its peak absorption at 692 nm, thus reflecting yellow light in acidic solutions, and the deprotonated form has its peak absorption at 602 nm, thus reflecting blue light in more basic solutions [79,80,81]. Figure 2 shows the results of the protonographic analysis, and the enzyme activity was detected as yellow bands against the blue background. The protonogram of BpsCAβ (Figure 2B) showed a 30 kDa band of activity. As described in the literature, mammal α-CAs are monomeric, and the protonogram of bCA showed a single band of activity corresponding to a monomer of 30 kDa. However, as mentioned previously, β-CAs are active enzymes for the CO_2_ hydratase reaction when assembled into dimers, tetramers, or octamers. The yellow bands found corresponding to the inactive monomeric form of BpsCAβ is due to the fact that at the end of the electrophoretic run, the SDS is removed from the gel. This procedure may lead to the rearrangement of BpsCAβ monomers in the gel, and the final result is the reconstitution of the active oligomeric forms of BpsCAβ.

### 2.2. Kinetic Constants

In Table 2 are shown the rate constants (k_cat_, K_M_, and k_cat_/K_M_) of the BpsCAβ identified in the genome of *Burkholderia pseudomallei* and the inhibition constant (K_I_) using the inhibitor acetazolamide. These constants were compared with the kinetic parameters of the α-CA from the *Homo sapiens* (isoforms hCA I and hCA II) and with those obtained from BpsCAγ that were reported previously [7]. The catalytic activity values of these enzymes were determined using the “stopped-flow” technique. BpsCAβ showed a k_cat_ of 1.6 × 10^5^ s^−1^ and a k_cat_/K_M_ of 3.4 × 10^7^ M^−1^·s^−1^. It was slightly less active than the human isoform hCAI (k_cat_ = 2.0 × 10^5^ s^−1^). Interestingly, the γ-CA from *B. pseudomallei* showed a k_cat_ = 5.3 × 10^5^ s^−1^, which is 3.3 times higher than the BpsCAβ (k_cat_ = 1.6 × 10^5^ s^−1^). This is also evident for the γ-CA from *V. cholerae*, which was 2.21 times more active than VchCAβ (k_cat_ = 7.39 × 10^5^ s^−1^). In addition, acetazolamide was found to be a less effective inhibitor for BpsCAβ (K_I_ = X nM) and BpsCAγ (K_I_ = Y nM) [48,49,50] than for hCA II, VchCAα, and hpβCA.

### 2.3. Sulfonamides and Sulfamates Inhibition Profiles

A panel of simple aromatic and heteroaromatic sulfonamides of types **1**–**24** and clinically used derivatives such as **AAZ**–**HCT** (Figure 1) was used to investigate the sulfonamide/sulfamate inhibition profile of BpsCAβ. Acetazolamide (**AAZ**), methazolamide (**MZA**), ethoxzolamide (**EZA**), and dichlorophenamide (**DCP**) are the classical systemically acting CAIs [51,52,53,54,55,56,57,58,59]. Dorzolamide (**DZA**) and brinzolamide (**BRZ**) are topically-acting anti-glaucoma agents; benzolamide (**BZA**) is an orphan drug belonging to this class of pharmacological agents; whereas topiramate (**TPM**), zonisamide (**ZNS**), and sulthiame (**SLT**) are widely used antiepileptic drugs [60,61,62,63,64,65]. Sulpiride (**SLP**), indisulam (**IND**), valdecoxib (**VLX**), celecoxib (**CLX**), and hydrochlorothiazide (**HCT**) were shown by these groups to belong to this class of pharmacological agents, acting as efficient inhibitors against many enzymes from mammals, plants, and microorganisms such as bacteria, protozoa, and fungi [66,67,68,69,70,71,72,73,74,75,76,77,78,79]. Sulfonamides **1**–**24** and the clinically used agents investigated in this study were either commercially available or were prepared as reported earlier by our group [21,25,42].

Inhibition data with the 39 investigated sulfonamides/sulfamates against the two enzymes from *B. pseudomallei* BpsCAβ and BpsCAγ, as well as the human main isoforms hCA I and II (as possible off targets) are shown in Table 3. The following structure–activity relationship (SAR) can be drawn from the data of this table:

(i) Some sulfonamides, including **1**, **4**, **6**–**9**, **18**, **19**, **24**, **SLP**, **VLX**, **CLX**, **SLT**, and the sulfamate **TPM** did not inhibit BpsCAβ significantly up to 50 µM concentration of inhibitor within the assay system (Table 3). They include a variety of chemotypes, such as simple benzenesulfonamides with compact *meta*- or *para*-substituents (**1**, **4**, **6**, **18**), halogen sulfanilamides (**7**–**9**), the compounds with elongated molecules **19** and **24**, as well as the clinically used compounds with a rather bulky scaffold such as the two coxibs, sulpiride and the sugar sulfamate **TPM**. It is thus difficult to draw a precise SAR for this limited number of inhibitors with such diverse structural features in their molecules.

(ii) A large number of the investigated sulfonamides were moderately potent inhibitors of BpsCAβ, with inhibition constants in the micromolar range. They include: **2**, **3**, **5**, **10**, **13**–**16**, **21**–**23**, **EZA**, **DZA**, **BRZ**, **ZNS**, **IND**, and **HCT** (Table 3), and their K_I_ range between 1500 and 5200 nM. Several interesting SAR data can be observed: the shift of the amino moiety from *meta* (as in **1**) to *para* (as in **2**) led to a net increase in the inhibitory action, as sulfanilamide **2** is moderately potent whereas metanilamide **1** was devoid of activity. The same activity was shown by **5**, which has an extra methylene moiety compared to sulfanilamide, but the increase of the linker to two methylene groups (as in **6**) leads to the complete loss of activity. It is also interesting to note that the introduction of halogens in the sulfanilamide **2** scaffold (as in **7**–**9**) led to a loss of activity against BpsCAβ, although the halogenated sulfonamides were more effective hCA I and II inhibitors compared to sulfanilamide itself (Table 3). For the phenol/alcohols **15**–**17** on the other hand, the increase of the linker between the OH moiety and the aromatic ring from 0 to 2 led to an increase of the BpsCAβ inhibitory action, with the best inhibitor being the hydroxyethyl derivative **17** (K_I_ of 417 nM). However, for the similar subseries **22**–**24**, the SAR is more complex, with **23** acting as the best inhibitor and the shorter or the longer molecule congeners (**22** and **24**) showing a decrease (or a loss) of activity (Table 3). The bulky clinically used derivatives **EZA**, **DZA**, **BRZ**, **ZNS**, **IND**, and **HCT** also belong to this category of CAIs with medium potency inhibitory action against BpsCAβ

(iii) The most efficient BpsCAβ inhibitors detected among the investigated sulfonamides were **11**, **12**, and **DCP** (belonging to the benzene-1,3-disulfonamide class of compounds), **17** (already mentioned above), **20**, and **BZA** (benzolamide-type CAIs possessing an elongated molecule and two sulfonamide functionalities), **AAZ**, and **MZA**. These sulfonamides showed K_I_ in the range of 185–745 nM (Table 3). The following SAR is obvious: the deacetylated derivatives of **AAZ** (**13**) and **MZA** (**14**) were much less effective BpsCAβ inhibitors compared to the clinically used drugs. This is a general phenomenon seen for many other CAs investigated earlier [65,66,67,68,69,70,71,72,73,74,75,76,77,78,79]. The best BpsCAβ inhibitors were **MZA** and **BZA**, with an almost identical inhibition constant (185–186 nM). It is interesting to note that **MZA**—which differs only by a CH_2_ moiety from **AAZ**—was four times stronger as an inhibitor than acetazolamide. Considering benzolamide (**BZA**), the introduction of a 4-amino moiety as in aminobenzolamide (**20**), led to a small decrease of the inhibitory activity from 185 nM to 266 nM (Table 3). Such compounds belong to a class of disulfonamide CAIs, which are highly effective against many CAs [80]. As mentioned above, the same efficient inhibitory action was observed for the benzene-1,3-disulfonamides **DCP**, **11**, and **12**, which incorporate two primary sulfonamide moieties in their scaffolds. Overall, the main conclusion is that several important CAI scaffolds lead to effective BpsCAβ inhibition, but compounds with an inhibition constant <100 nM were not identified in this study.

(iv) The inhibition profile of BpsCAβ differs substantially from that of the γ-class enzyme from the same pathogen (BpsCAγ) that was recently investigated by this group [7]. A relatively limited number of sulfonamides that have been investigated act as effective CAIs against both the β- and γ-CAs from this bacterium (Table 3). Among them are **20**, **AAZ**, **MZA**, and **BZA**, with the latter compound having the most promising profile (K_I_ of 653 and 185 nM, respectively). Thus, **BZA** might be considered an important lead compound for obtaining more effective CAIs targeting *Burkholderia pseudomallei*.

## 3. Materials and Methods

### 3.1. Gene Identification and Cloning

The identification of the gene encoding *B. pseudomallei* β-CA (BpsCAβ) was performed as described by Del Prete et al. [61] Briefly, the β-CA gene of *B. pseudomallei* with the accession number WP_004189176.1 was identified running the ‘‘BLAST’’ program, using the nucleotide sequences of bacterial β-CAs as query sequence. The GeneArt Company (Invitrogen)—specializing in gene synthesis—designed the synthetic BpsCAβ gene (BpsCAβ-DNA) encoding for the BpsCAβ (protein of 256 amino acid residues) containing four base-pair sequences (CACC) necessary for directional cloning at the 5′ end of the PfCAdom gene. The recovered PfCAdom gene and the linearized expression vector (pET-100/D-TOPO) were ligated by T4 DNA ligase to form the expression vector pET-100/BpsCAβ.

### 3.2. Expression and Purification of the Recombinant BpsCAβ

BL21-CodonPlus(DE3)-RIPL competent cells (Agilent) were transformed with pET-100/BpsCAβ, grown at 37 °C, and induced with 1 mM isopropil-β-d-1-tiogalattopiranoside (IPTG). After 30 min, ZnSO_4_ (0.5 mM) was added to the culture medium and cells were grown for an additional 3 h. Subsequently, cells were harvested and re-suspended in the following buffer: 50 mM Tris/HCl, pH 8.0, 0.5 mM phenylmethylsulfonyl fluoride (PMSF), and 1 mM benzamidine. Cells were then disrupted by sonication at 4 °C. After centrifugation at 12,000× *g* for 45 min, the supernatant was incubated with His Select HF nickel affinity gel resin (Sigma) equilibrated in lysis buffer for 30 min. Following centrifugation at 2000× *g*, the resin was washed in buffer (50 mM Tris/HCl, pH 8.3, 500 mM KCl, 20 mM imidazole). The protein was eluted with the wash buffer containing 300 mM imidazole. The collected fractions were dialyzed against 50 mM Tris/HCl, pH 8.3. At this stage of purification, the protein was at least 95% pure and the obtained recovery was of about 20 mg of the recombinant protein.

### 3.3. Protonography

Sodium dodecyl sulfate (SDS)-polyacrylamide gel electrophoresis (PAGE) was performed as described by Laemmli [83]. Wells of 12% SDS-gel were loaded with bCA or BpsCAβ mixed with Laemmli loading buffer without 2-mercaptoethanol and without boiling the samples in order to avoid protein denaturation. The gel was run at 180 V until the dye front ran off the gel. Following the electrophoresis, the gel was removed from glass plates and soaked in 2.5% Triton X-100 for 1 h on a shaker and then twice in 100 mM Tris, pH 8.2 containing 10% isopropanol for 10 min. Subsequently, the gel was incubated in 0.1% bromothymol blue in 100 mM Tris, pH 8.2 for 30 min and then immersed in CO_2_-saturated ddH_2_O to visualize the hydratase activity of the enzyme. The assay was performed at room temperature and the CO_2_-saturated solution was prepared by bubbling CO_2_ into 200 mL distilled water for approximately 3 h. The local decrease in pH due to the presence of CA activity was evidenced by the formation of yellow bands due to the change of the indicator color from blue (alkaline pH) to yellow (acidic pH) [79,80,81].

### 3.4. Kinetic and Inhibition Assay

An Applied Photophysics stopped-flow instrument has been used for assaying the CA-catalyzed CO_2_ hydration activity. [82] Phenol red (at a concentration of 0.2 mM) was used as indicator, working at the absorbance maximum of 557 nm, with 20 mM TRIS (pH 8.3) as buffer, and 20 mM NaClO_4_ (for maintaining constant the ionic strength), following the initial rates of the CA-catalyzed CO_2_ hydration reaction for a period of 10–100 s. The CO_2_ concentrations ranged from 1.7 to 17 mM for the determination of the kinetic parameters (by Lineweaver–Burk plots) and inhibition constants. For each inhibitor, at least six traces of the initial 5%–10% of the reaction were used to determine the initial velocity. The uncatalyzed rates were determined in the same manner and subtracted from the total observed rates. Stock solutions of inhibitor (10–100 mM) were prepared in distilled-deionized water, and dilutions up to 0.01 mM were done thereafter with the assay buffer. Inhibitor and enzyme solutions were preincubated together for 15 min at room temperature prior to assay in order to allow for the formation of the E-I (Enzyme-Inhibitor) complex or for the eventual active site-mediated hydrolysis of the inhibitor. The inhibition constants were obtained by non-linear least-squares methods using PRISM 3 and the Cheng–Prusoff equation (as reported earlier), and represent the mean from at least three different determinations. All CA isoforms were recombinant ones obtained in-house. All salts/small molecules were of the highest purity available, from Sigma-Aldrich (Milan, Italy).

## 4. Conclusions

We have cloned, purified, and characterized a β-CA from the pathogenic bacterium *Burkholderia pseudomallei*, responsible for the tropical disease melioidosis, named here BpsCAβ. The enzyme showed a relatively high catalytic activity for the physiologic CO_2_ hydration reaction to bicarbonate and protons, with the following kinetic parameters: k_cat_ of 1.6 × 10^5^ s^−1^ and k_cat_/K_M_ of 3.4 × 10^7^ M^−1^·s^−1^. An inhibition study with a panel of 38 sulfonamides and one sulfamate—including many agents that are used clinically—revealed an interesting structure–activity relationship for the interaction of this enzyme with its inhibitors. Many simple sulfonamides and clinically used agents such as topiramate, sulpiride, celecoxib, valdecoxib, and sulthiame were ineffective BpsCAβ inhibitors (K_I_ > 50 µM). Other drugs, such as ethoxzolamide, dorzolamide, brinzolamide, zonisamide, indisulam, and hydrochlorothiazide were moderately potent micromolar inhibitors. The most promising inhibition was observed using benzene-1,3-disulfonamides, benzolamide and its analogs, acetazolamide, and methazolamide, which showed K_I_ in the range of 185–745 nM. The inhibition profile of BpsCAβ differs substantially from that of the γ-class enzyme from the same pathogen (BpsCAγ), making it rather difficult to propose compounds that would effectively interact with both enzymes. However, benzolamide was one of the most promising inhibitors of both these CAs, with K_I_ of 653 and 185 nM, respectively. Thus, benzolamide is an interesting lead compound for the design of more effective such agents, which may be useful tools for understanding the pathogenicity of this bacterium.

## Figures and Tables

**Table 1:** Bacterial carbonic anhydrases (CAs) investigated by our groups for their biochemical properties, kinetic constants, and inhibition profiles.

Bacterium	CA		Characteristics		Inhibition	
	Class	Acronym	Pathogenicity	Disease	Sulfonamides	Anions
*Vibrio cholerae* [8,49,50]	α	VchCAα	+	Cholera	+	+
	β	VchCAβ			+	+
	γ	VchCAγ			+	+
*Helicobacter pylori* [36,51,52]	α	hpαCA	+	Gastritis, gastric	+	+
	β	hpβCA		ulcers	+	+
*Streptococcus mutans* [11,53,54]	β	SmuCA	+	Caries	+	+
*Haemophilus influenzae*	β	HICA	+	Influenza	−	+
*Neisseria gonorrhoeae*	α	NgoCA	+	Gonorrhea	+	+
*Neisseria sicca*	α	NsiCA	+	Septicemia	+	−
*Porphyromonas gingivalis* [11,13,55,56,57]	β	PgiCA β	+	Periodontitis,	+	+
	γ	PgiCA γ		rheumatoid arthritis	+	+
*Legionella pneumophila* [42]	β	LpCA1	+	Legionellosis	+	+
	β	LpCA2			+	+
*Clostridium perfringens* [58]	β	CpeCA	+	Food poisoning	−	+
*Brucella suis* [34,59,60]	β	bsCA 1	+	Brucellosis	+	−
	β	BsCA II			+	−
*Burkholderia pseudomallei* [1,7,61]	β	BpsCAβ	+	Melioidosis	−	+
	γ	BpsCAγ			+	+
*Salmonella enterica* [62,63]	β	stCA 1	+	Salmonellosis	+	+
	β	stCA 2			+	+
*Streptococcus pneumoniae* [64]	β	PCA	+	Pneumonia	+	+
*Mycobacterium tuberculosis* [65,66,67]	β	mtCA 1	+	Tuberculosis	+	−
	β	mtCA 2			+	−
	β	mtCA 3			+	−
*Methanobacterium thermoautotrophicum* [68]	β	Cab	−	−	+	+
*Methanosarcina thermophila* [69]	γ	Zn-Cam	−	−	+	+
	γ	Co-Cam	−	−	+	+
*Sulfurihydrogenibium yellowstonense* [19,70,71,72]	α	SspCA	−	−	+	+
*Sulfurihydrogenibium azorense* [73,74,75,76]	α	SazCA	−	−	+	+
*Colwellia psychrerythraea* [10,38]	γ	CpsCA	−	−	+	+
*Pseudoalteromonas haloplanktis* [77,78]	γ	PhaCAγ	−	−	+	+

**Table 2:** Kinetic parameters for the CO_2_ hydration reaction catalysed by the human cytosolic isozymes hCA I and II (α-class CAs) at 20 °C and pH 7.5 in 10 mM HEPES buffer and 20 mM Na_2_SO_4_, β- and γ-CAs from *B. pseudomallei* measured at 20 °C, pH 8.3 in 20 mM TRIS buffer and 20 mM NaClO_4_ [82], α-, β-, and γ-CAs from *Vibrio cholerae* [8,49,50], and β-CA from *Helicobacter pylori* [36,51,52]. Acetazolamide inhibition data are also shown.

Enzyme	Activity Level	Class	k_cat_ (s^−1^)	k_cat_/K_m_ (M^−1^·s^−1^)	K_I_ (Acetazolamide) (nM)
hCA I	moderate	α	2.0 × 10^5^	5.0 × 10^7^	250
hCA II	very high	α	1.4 × 10^6^	1.5 × 10^8^	12
BpsCAβ	moderate	β	1.6 × 10^5^	3.4 × 10^7^	745
BpsCAγ	moderate	γ	5.3 × 10^5^	2.5 × 10^7^	149
VchCAα	high	α	8.23 × 10^5^	7.0 × 10^7^	6.8
VchCAβ	moderate	β	3.34 × 10^5^	4.1 × 10^7^	451
VchCAγ	high	γ	7.39 × 10^5^	6.4 × 10^7^	473
hpβCA	high	β	7.1 × 10^5^	4.8 × 10^7^	40

**Table 3:** Sulfonamides/sulfamates inhibition constants (K_I_, nM) for the human α-CAs (isoforms hCA I and II) and the β-/γ-CAs identified in the genome of *B. pseudomallei*, assayed by a CO_2_ hydrase stopped flow method [7,50].

Inhibitor	K_I_, nM *			
	hCA I	hCA II	BpsCAγ	BpsCAβ
**1**	45,400	295	574	>50,000
**2**	25,000	240	1720	3895
**3**	28,000	300	1550	3170
**4**	78,500	320	>50,000	>50,000
**5**	25,000	170	>50,000	3900
**6**	21,000	160	>50,000	>50,000
**7**	8300	60	>50,000	>50,000
**8**	9800	110	12,500	>50,000
**9**	6500	40	>50,000	>50,000
**10**	6000	70	>50,000	4100
**11**	5800	63	14,000	425
**12**	8400	75	23,500	307
**13**	8600	60	18,400	3065
**14**	9300	19	1810	2500
**15**	6	2	9650	4345
**16**	164	46	10,800	2215
**17**	185	50	1825	417
**18**	109	33	1500	>50000
**19**	95	30	1838	>50000
**20**	690	12	1810	266
**21**	55	80	1335	1650
**22**	21,000	125	1805	5200
**23**	23,000	133	1700	1500
**24**	24,000	125	24,500	>50,000
**AAZ**	250	12	149	745
**MZA**	50	14	1595	186
**EZA**	25	8	1865	3850
**DCP**	1200	38	>50,000	529
**DZA**	50,000	9	2260	3670
**BRZ**	45,000	3	1270	4270
**BZA**	15	9	653	185
**TPM**	250	10	3010	>50,000
**ZNS**	56	35	>50,000	4060
**SLP**	1200	40	5600	>50,000
**IND**	31	15	1800	4375
**VLX**	>50,000	43	>50,000	>50,000
**CLX**	50,000	21	>50,000	>50,000
**SLT**	374	9	8900	>50,000
**HCT**	328	290	>50,000	3490

* Mean from three different assays. Errors in the range of ±10% of the reported values (data not shown).
